# Electrochemical Assessment of Anticancer Compounds on the Human Tongue Squamous Carcinoma Cells

**DOI:** 10.3390/s20092632

**Published:** 2020-05-05

**Authors:** Chun-Chung Huang, Tse-Hua Tung, Chien-Chu Huang, Shao-Yi Lin, Shih-Chi Chao, Sheng-Po Chiu, Shiao-Pieng Lee, Chun-Min Lo

**Affiliations:** 1 Department of Biomedical Engineering, National Yang-Ming University, Taipei 11221, Taiwan; dk800108@ym.edu.tw (C.-C.H.); d40004001@ym.edu.tw (T.-H.T.); 2 Graduate Institution of Biomedical Sciences, China Medical University, Taichung 40402, Taiwan; akamarucoh1116@gmail.com; 3 Department of Obstetrics and Gynecology, China Medical University Hospital, Taichung 40447, Taiwan; 4 Department of Mechanical and Computer-Aided Engineering, National Formosa University, Yunlin 63201, Taiwan; sgi.shaoyi@gmail.com; 5 Graduate Institute of Life Sciences, National Defense Medical Center, Taipei 11490, Taiwan; 805302029@mail.ndmctsgh.edu.tw; 6 Division of Endocrinology and Metabolism, Department of Internal Medicine, Tri-Service General Hospital Songshan Branch, National Defense Medical Center, Taipei 11490, Taiwan; shengpo.chiu@gmail.com; 7 Division of Oral and Maxillofacial Surgery, Department of Dentistry, School of Dentistry, Tri-Service General Hospital, National Defense Medical Center, Taipei 11490, Taiwan

**Keywords:** andrographolide, cannabidiol, cisplatin, fluorouracil, electric cell substrate impedance sensing (ECIS), oral cancer, SCC-25

## Abstract

The most common oral cancer is squamous cell carcinoma (SCC) and its highest occurrence is in the tongue. Almost 30% of patients with one primary head and neck tumor will have a second primary malignancy. In recent studies, two novel plant extracts, andrographolide and cannabidiol (CBD), have been exploited for their anticancer effects. Here, we investigated the cytotoxic effects of these two compounds on SCC-25 cells, a human tongue squamous carcinoma cell line, and compared the outcomes with two chemotherapeutic drugs, cisplatin and fluorouracil. Electric cell substrate impedance sensing (ECIS) system was applied to measure frequency- and time-dependent impedance of SCC-25 cell-covered electrodes and to further assess subtle changes in cell morphology and micromotion in response to different concentrations (0, 10, 30, 100, and 300 µM) of these compounds. AlamarBlue and Annexin V/7-AAD binding assays were used to measure the concentration dependent changes in viability and apoptosis of SCC-25 cells. Our results demonstrate that 24 hours after exposure to 30 µM CBD can significantly decrease the micromotion rate, damage the integrity of cell morphology, reduce cell viability, and induce higher apoptosis in treated SCC-25 cells, while the other three drugs attain similar effects at the concentration of 100 µM or higher. The apoptosis-induced changes in cell morphology and micromotion monitored by ECIS correlate well with biochemical assays. Thus, both frequency- and time-dependent impedance measurements using ECIS can be used to real-time follow cancer cell activities in response to anticancer drugs with different temporal cytotoxicity profiles.

## 1. Introduction

Human tongue squamous cell carcinoma (SCC) encompasses at least 90% of all oral malignancies and has the lowest five years survival rate among oral cancer patients [1,2]. This form of cancer exhibits frequent regional invasion and high propensity to metastasize to other sites of the body. Standard treatments consist of surgical resection followed by post-operation chemotherapy or radiotherapy [3,4]. According to Cathay Pacific Hospital and Taipei City United Hospital data bank, the cure fraction for stage I oral cancer is 80%, stage II is 60%–70%, stage III and IV are about 30%–50%. Despite advances in the new treatment modalities, patients still require intensive care and follow-up. Current chemotherapeutic medication inhibits tumor growth by disrupting cell cycle progression leading to cell apoptosis. Although they are standard procedures for cancer therapy, they are limited by toxicity or acquired resistance, which may lead to other side effects. To obtain an effective anticancer drug targeting the earlier stages of migration in the disease, development is desirable as it can inhibit the tumors and reduce metastasis.

Two novel plant extracts, cannabidiol (CBD) and andrographolide (Andro), have been exploited for their anticancer effects and more importantly for their non-toxic effects [5,6,7,8,9,10]. CBD is a phytocannabinoid that belongs to the cannabinoid family. It is extracted from *Cannabis sativa* and does not exhibit any psychotropic effects observed in ∆-9-tetrahydrocannabinol (THC) [5,11,12]. Cannabinoids have been reported to modulate signaling pathways central to the growth and spread of cancer. They can inhibit cell cycle progression and decrease cell migration [13,14]. CBD is reportedly effective to reduce tumor growth, as observed in animal models [5,11]. Furthermore, it has been reported to induce apoptosis in cancer cells through activation of classical caspase pathways [15]. The second extract explored, andrographolide, is a diterpenoid lactone extracted from *Andrographis paniculata* [9,10]. Previous research has shown andrographolide to suppress cell motility and proliferation [16]. Furthermore, the active ingredients have been shown to inhibit translocation of DNA [17], have high anti-inflammatory activity [18,19], and regulate the immune response as well as having some other effects. Substantial research of andrographolide has shown that it plays an extensive role in apoptosis through different signaling pathways [20,21].

Most of the cytotoxic anticancer drugs in use induce apoptosis in cancer cells and are selected on the basis of animal screening systems. The in vitro apoptotic responses of cancer cells induced by anticancer drugs may therefore be valuable predictors of their responses to these drugs in vivo. Morphologically, apoptotic cells share a number of common features including loss of focal adhesions, cytoplasmic shrinkage and nuclear condensation, membrane blebbing, and the formation of apoptotic bodies [22]. These morphological changes have been suggested to be an early prerequisite to apoptotic events leading to cell death [23,24]. In these studies, in addition to light and fluorescence microscopy, coulter-type cell size analyzer and atomic force microscopy (AFM) have been applied to observe morphological changes during apoptosis, particularly apoptotic volume decrease [22,23,24].

Electric cell-substrate impedance sensing (ECIS), a label-free and real-time electrochemical method, can be applied to measure subtle changes of cell morphology and micromotion in tissue culture [25,26,27,28]. By culturing cells on small gold film electrodes and monitoring impedance changes caused by adherent cells, changes in the capacitance of the cell membrane, cell-substrate separation, and cell–cell separation can be quantified with exquisite sensitivity and in a non-invasive manner. In ECIS the impedance of the cell-covered electrode increases after the attachment and spreading of cells. Fluctuations in the measured impedance time series are always related to living cells and observed even if the attached cells are confluent. Since electric currents flowing out of the electrode pass through the narrow space between cells and their substrate and then through the space between cells, these fluctuations are substantially associated with the subtle changes of cell morphology and have been referred to as micromotion, an indication of cell viability and motility [26,27].

ECIS has been applied to monitor morphological changes of adherent cells in response to a variety of stimuli under physiological and pathological conditions [29,30,31]. In particular, the time course of apoptosis-induced morphological changes of porcine brain capillary endothelial cells was monitored using ECIS and the disassembly of barrier-forming tight junctions was observed [32]. In this study, the apoptotic effects of SCC-25 cells after exposure to four different anticancer drugs, andrographolide (Andro), cannabidiol (CBD), cisplatin (Cis), and fluorouracil (5-FU) were examined. The subsequent morphological changes and cellular micromotion associated with apoptosis were monitored by ECIS. Biochemical assays, such as alamarBlue and Annexin V/7-AAD binding assays and western blotting analysis were also performed to correlate the ECIS data. Our results demonstrate that CBD presents significant effect in inhibiting SCC-25 migration and motility. A combination of morphological biosensors like ECIS and biochemical techniques that measure anticancer drug effects is likely to offer reliable data for evaluating chemotherapeutic agents.

## 2. Materials and Methods

### 2.1. Cell Preparation and Culture Conditions

The tongue squamous cell carcinoma (SCC-25) were obtained from Bioresource Collection and Research Centre in Taiwan (BCRC No.60516). SCC-25 cells were cultured in a 1:1 ratio of Dulbecco’s Modified Eagle’s medium and Ham’s F12 medium containing 1.5 g/L sodium bicarbonate, 2.5 mM L-glutamine, 15 mM HEPES, 0.5 mM sodium pyruvate, supplemented with 90% 400 ng/mL hydrocortisone and 10% fetal bovine serum under 5% CO_2_ at 37 °C. The medium was changed every two days. Cells were cultured for cell passage or experiment when they reached 80% confluency.

### 2.2. Impedance Measurement by ECIS

The ECIS ZӨ system and components were obtained from Applied BioPhysics (Troy, NY, USA). The 8W1E arrays (8 wells with a 250 μm diameter gold sensing electrode) were used in this study. In ECIS, the interaction between cell and substrate is measured via the electrodes that are connected through the relay bank to a lock-in amplifier which measured in- (real) and out-of-phase (imaginary) voltages across the cell-covered electrode. Each well was seeded with cell density of 1.25 × 10^5^ cells/cm^2^. Confluency was reached 24 h after seeding and the cells were treated with each of the four different anticancer drugs for an additional 24 h. To quantify the changes in cell morphology, impedance of each cell-covered electrode was measured at 25 different frequencies, ranging from 31.25 Hz to 100 kHz. In general, impedances for both a cell-free electrode and the same electrode covered with SCC-25 cells were measured. These kinds of frequency-scan measurements were applied before and after cells were exposed to different concentrations of anticancer compounds. By comparing the experimental data of cell-covered electrode with the calculated values obtained from a suitable cell-electrode model, morphological parameters such as junctional resistance between adjacent cells (Rb), average separation distance between basolateral cell surface and substratum (h), and capacitance of the cell membrane (Cm) can be obtained [26,28]. For detection of cell micromotion, impedance time series data of each electrode well were taken every second at 4 kHz until 2048 points were acquired. In order to compare the different level of impedance fluctuations obtained under the influence of different compounds and concentrations, variance for the 32-point sections (Var32) was used. In this numerical analysis, each data point in the 2048-point data set was first divided by their average value. Var32 was obtained by taking the average of the 64 variance values which were calculated from each 32-point section of the normalized 2048 data points.

### 2.3. Cell Viability Assay

Cell viability was quantified by alamarBlue assay. After SCC-25 cells were treated for 24 h with andrographolide, CBD, cisplatin, and 5-FU at different concentrations (0, 10, 30, 100, and 300 µM), the viability reagent was added into each well. The plates were incubated for 4 h at 37 °C and then read at 570 nm and 600 nm. Cell viability was calculated according to the following formula: reduction of alamarBlue reagent = [(experimental RFU value − negative control RFU value)/(100% reduced positive control RFU value − negative control RFU value)] × 100.

### 2.4. Annexin V/7-AAD Binding Assay

Annexin V/7-AAD staining method was used to identify and quantify apoptotic cells. SCC-25 cells (2 × 10^5^–5 × 10^5^ cells/mL) were treated according to the manufacturer’s instruction of ANNEX V Kit. The ability of different treatments to induce apoptosis against SCC-25 cells was assessed by flow cytometry using the Muse^®^ Annexin V and Dead Cell Assay kit (EMD Millipore Corporation). The extent of apoptosis was quantified according to the percentage of Annexin V-positive cells. The ability of the different treatments to induce apoptosis against tongue squamous carcinoma cells was assessed for this analysis. SCC-25 cells were plated in 24-well plates at a density of 5 × 10^4^ cells per well. After 24 h of incubation, cells were incubated with various concentrations of CBD, andrographolide, cisplatin, and 5-FU (at 0, 10, 30, 100, and 300 µM) for 24 h. After 24 h of treatment, cells were detached by trypsin and then centrifuged at 1200 rpm for 10 min. After centrifugation, 1 × 10^5^ cells were stained according to the manufacturer’s instructions and analyzed using Guava easyCyte Plus Flow Cytometer (Millipore, Billerica, MA, USA).

### 2.5. Statistical Analysis

Significant differences between the control and each of the experimental groups was performed using one-way ANOVA. Once the significance of the group differences (*p* < 0.05) was determined, post hoc Tukey’s tests were subsequently used for pairwise comparisons. The level of significance was set at * *p* < 0.05, ** *p* < 0.01, and *** *p* < 0.001. All data are expressed as mean ± standard error of the mean.

## 3. Results

### 3.1. Effects of Anticancer Compounds on the Overall Resistance Time Course of SCC-25 Cells

**Figure 1:**
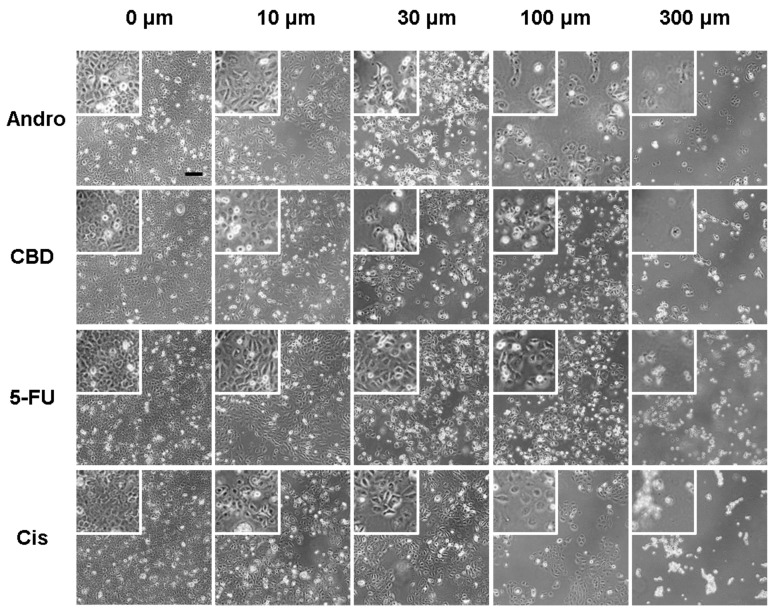
Typical phase-contrast images of SCC-25 cells cultured in 24-well cell culture plates 24 h after exposure to different concentrations (0, 10, 30, 100, and 300 µM) of anticancer compounds, andrographolide (Andro), CBD, 5-FU, and cisplatin (Cis). Five independent experiments were performed (n = 5). Cell number decreases as the compound concentration increases. Cells exhibit perceptibly distressed and shrunken morphology 24 h after exposure to 100 µM or 300 µM of any of the four anticancer compounds. Scale bar = 200 µm.

SCC-25 cells were treated with different concentrations of andrographolide, CBD, 5-FU, and cisplatin (0, 10, 30, 100, and 300 µM). Morphological changes associated with apoptosis were observed by phase contrast images after 24 h exposure to the anticancer drugs (Figure 1). As the concentration of each compound increased, all the cells became rounded, shrunken, and eventually detached from the substrate after the treatment, all of which are essential features of apoptotic cells. However, the subtle changes of cell morphology were not clear in these optical images.

**Figure 2:**
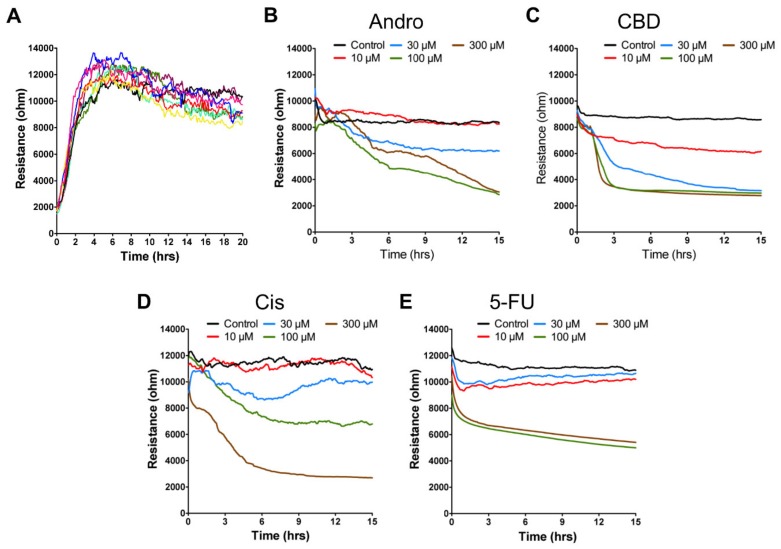
(**A**) Resistance time series of SCC-25 cell attachment and spreading. Each colored curve represents the time course obtained from each of the eight electrode-containing wells. Typical resistance time course of confluent SCC-25 cell layers upon addition of various concentrations of (**B**) andrographolide, (**C**) CBD, (**D**) 5-FU, and (**E**) cisplatin. Three independent ECIS experiments were performed (n = 3). In each experiment, three wells were measured for each drug concentration. At time 0, each anticancer compound dissolved in DMSO was added to give the final concentrations of 10 μM (red), 30 μM (blue), 100 μM (green), 300 μM (brown), and control (black), and the resulting changes in resistance were followed. Data were collected for 15 h after adding for each compound.

In a parallel experiment, morphological changes of SCC-25 cells were measured by ECIS during the anticancer drug treatment. First, SCC-25 cell attachment and spreading on the sensing electrode was monitored. Impedance data were measured at 11 different frequencies (62.5–64 kHz) from time 0, right after the cells were inoculated into electrode wells, to 20 h after cell layers were confluent. Here, only the resistance (real part of the impedance) time course measured at 4 kHz is shown (Figure 2A). The cell-free resistance is about 2 kΩ in each well. As cells attach and spread onto the electrodes, they effectively block the current flow and a large increase in the impedance was observed. A confluent layer generally reaches the highest resistance value at 5 h after inoculation. As seen in Figure 2A, for SCC-25 cells the resistance changes as a function of time exhibits a large increase in the measured resistance, approximately 10–12 kΩ. Smaller changes were observed due to cellular micromotion that caused impedance to fluctuate with time.

Figure 2B–E show the typical resistance time course of confluent SCC-25 cell layers challenged by adding various concentrations (0, 10, 30, 100, and 300 μM) of andrographolide, CBD, 5-FU, and cisplatin, respectively. The effect of each compound on the overall resistance of the MCC-25 cell layers was monitored for 15 h. At high concentrations such as 100 or 300 μM, a rapid drop of resistance was observed almost immediately following the addition of CBD or 5-FU, whereas a slower decline of resistance was observed following the addition of andrographolide or cisplatin. At low concentrations such as 0, 10, and 30 μM, the resistance drop was less evident or absent except at 30 μM of CBD. Though the resistance time course indicates the overall morphological changes of cells, more quantitative methods are needed to analyze the different cytotoxic effects of these four compounds on SCC-25 cells.

### 3.2. Effects of Anticancer Compounds on the Morphological Parameters of SCC-25 Cells

**Figure 3:**
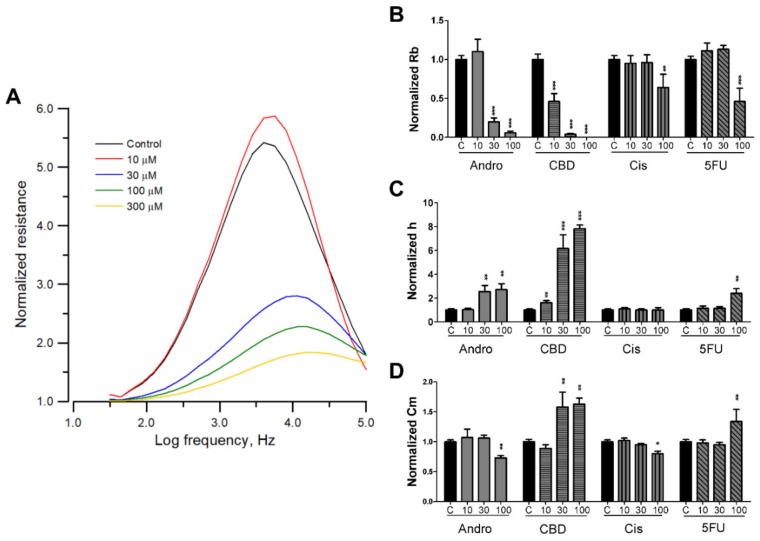
(**A**) Normalized resistance curves as a function of frequency obtained from a frequency-scan measurement 24 h after the addition of andrographolide to confluent SCC-25 cell layers at various concentrations of 10 μM (red), 30 μM (blue), 100 μM (green), 300 μM (yellow), and control (black). Changes in morphological parameters of confluent SCC-25 cell layers upon challenge with different concentrations of andrographolide, CBD, 5-FU, and cisplatin, respectively. Normalized junctional resistance between cells (**B**), average cell–substrate separation (**C**), and membrane capacitance (**D**) of the SCC-25 cell layers were obtained through cell-electrode model calculation of the frequency-scan data. Six independent frequency scan experiments were performed (n = 6). In each experiment, two or three wells were measured for each drug concentration. Values shown are mean ± standard error. * *p* < 0.05, ** *p* < 0.01, *** *p* < 0.001. Each value is expressed as a percentage of control.

To further understand the cytotoxic effects of andrographolide, CBD, 5-FU, and cisplatin on morphological changes of SCC-25 cells—especially on cell–cell and cell–substrate contacts—frequency-scan measurements were taken from cell-covered electrodes 24 h after exposure to different concentrations of each compound. Normalized resistance as a function of frequency was calculated by dividing the cell-covered resistance measured at various frequencies with the corresponding cell-free value (Figure 3A). For the normalized resistance spectrum of the control cells (black lines in Figure 3A), the ratio value started from about 1.1 at 31.5 Hz, increased with increasing frequency to the peak value, approximately 5.5 at 4 kHz, and then decreased with increasing frequency until about 1.8 at 100 kHz. This biphasic feature of the normalized resistance spectrum indicates that impedance measurement at 4 kHz can sensitively reflect the morphological changes of SCC-25 cells. After fitting the frequency-scan data with the cell-electrode model calculation, the junctional resistance between cells (Rb), average cell–substrate separation (h), and membrane capacitance (Cm) of the control SCC-25 cells were 1.8 Ω·cm^2^, 34 nm, and 2.5 μF/cm^2^, respectively. These are typical parameter values for many endothelial or epithelial cell lines.

Morphological parameters in response to each drug concentration shown in Figure 3B–D are expressed as percentages of control for easy comparison. Since many SCC-25 cells detached after 24 h exposure to 300 μM of any tested drug, the model analysis was unable to fit the frequency-scan data at this concentration. The result shown in Figure 3 indicates that upon challenge of 0, 10, 30, and 100 μM CBD, Rb decreases and h increases in a dose-dependent manner. In addition, while Cm is not changed 24 h after 10 μM CBD treatment, Cm values at 30 and 100 μM CBD are approximately 1.5-fold larger than control, implying roughed and wrinkled cell membrane upon treatment. Compared with CBD result, exposure of SCC-25 cells to 30 and 100 μM andrographolide, 100 μM cisplatin, and 100 μM 5-FU for 24 h showed less significant effect on Rb, h, and Cm values. SCC-25 cells were not significantly affected by 30 μM cisplatin or 30 μM 5-FU.

### 3.3. Effects of Anticancer Compounds on the Cellular Micromotion of SCC-25 Cells

**Figure 4:**
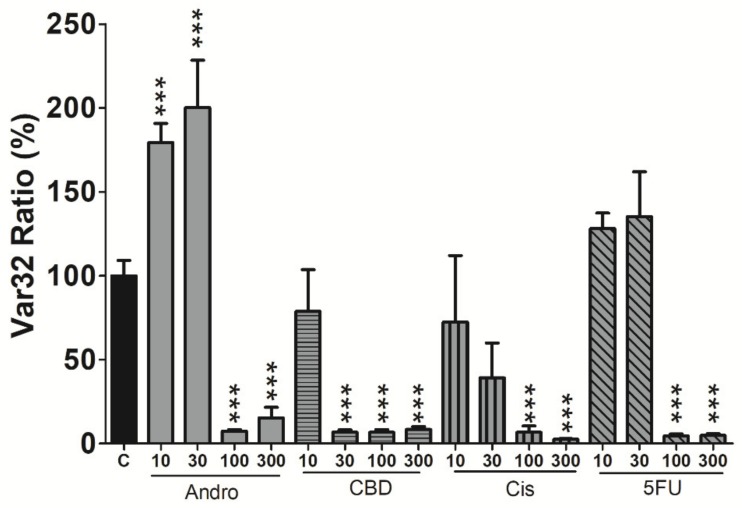
Var32 analysis of SCC-25 cell micromotion 24 h after exposure to different concentrations of each compound. Values shown are mean ± standard error (n = 4 for each concentration). * *p* < 0.05, ** *p* < 0.01, *** *p* < 0.001. Each value is expressed as a percentage of control.

SCC-25 cell micromotion was measured 24 h after exposure to different concentrations of each compound. In this assay, impedance data were taken every second at 4 kHz for 2048 s and subtle fluctuations in the resistance time course can be sensitively detected (Figure S1). It is important to note that the average value of the resistance time course under the treatment of higher concentration drug is smaller than that of lower concentration. Thus, it seems appropriate to normalize the data by dividing each data point with their average value before calculating the fluctuations. Numerical analysis was then applied to characterize the normalized resistance time courses and Var32 values were calculated. The decreased fluctuations for the drug-treated cells, as compared with control cells, generally represents decreased micromotion. As shown in Figure 4, there was a drastic impairment of cell micromotion in response to the challenge of each compound at 100 and 300 μM. For the lower concentration at 30 μM, only CBD treatment showed the evident reduction of micromotion. However, Var32 values of andrographolide-treated cells at 10 and 30 μM were larger than control cells.

### 3.4. Effects of Anticancer Compounds on Cell Viability

**Figure 5:**
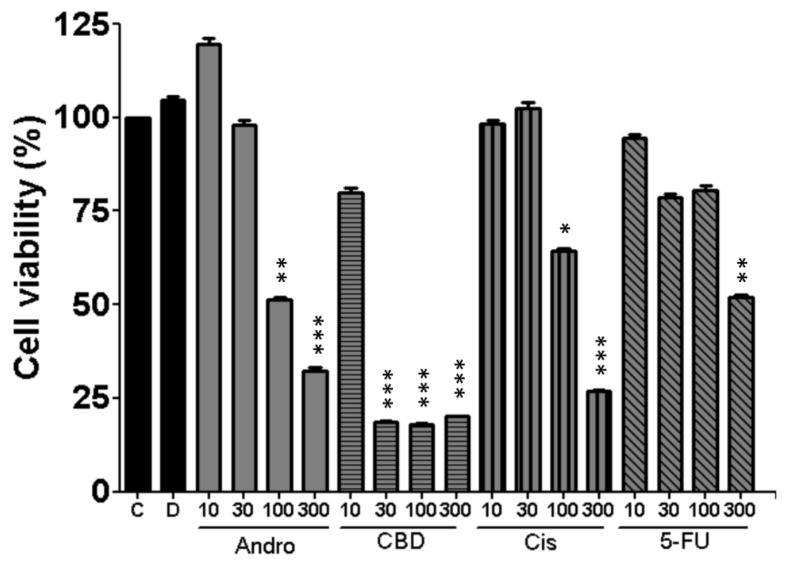
Change of cell viability of SCC-25 cells upon treatments of andrographolide, CBD, cisplatin and 5-FU for 24 h at the indicated concentrations. In each experiment there was a control (C) and cells treated with DMSO (D). Cell viability was evaluated by alamarBlue assay. All treatments at higher concentrations displayed statistically significant reductions relative to the control groups. Five independent experiments were performed (n = 5) and results are shown as mean ± SEM. * *p* < 0.05, ** *p* < 0.01, *** *p* < 0.001.

The cytotoxic effects of andrographolide, CBD, cisplatin and 5-FU on SCC-25 cells were evaluated using alamarBlue assay. As shown in Figure 5, both control and DMSO groups displayed non-toxicity effect on the cells, maintaining the same values for all experiments. A decrease in cell viability was observed upon increasing the concentration of all drugs and the highest toxicity was found to be 100 and 300 µM. Compared with the other three drugs, CBD exhibited a more significant decreased viability at lower concentration of 30 μM. Therefore, SCC-25 cells seem to be more sensitive to CBD than the other three drugs.

### 3.5. Apoptosis Profile of SCC-25 Cells Induced by Drug Treatment

**Figure 6:**
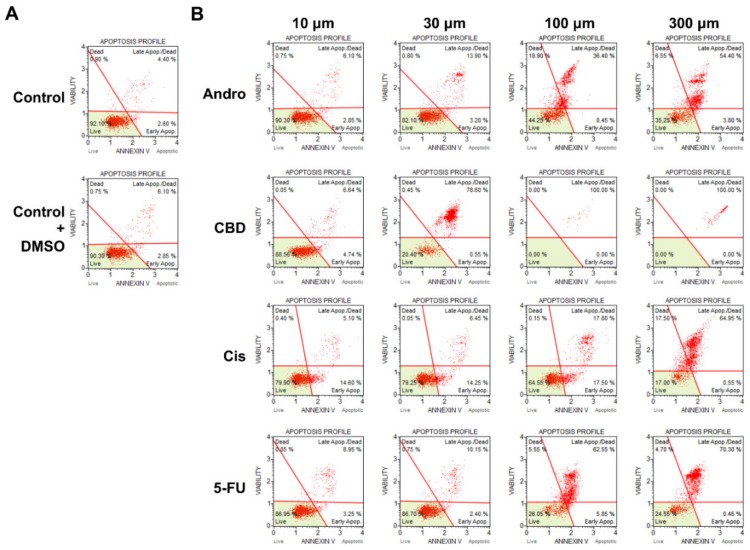
Apoptosis analyzed by flow cytometry following Annexin V/7-AAD binding assays. (**A**) Two negative control groups were used: cells without drug treatment and cells treated with DMSO. (**B**) SCC-25 cells were treated for 24 h at four different concentrations of andrographolide, CBD, cisplatin, and 5-FU.

To further distinguish the different stages of apoptosis induced by the four drugs, SCC-25 cells were treated with each drug at different concentrations and analyzed by the Annexin V/7-AAD binding assay. As seen in Figure 6, treatment with 100 µM andrographolide resulted in 36.40% of cells in the late apoptotic stage, whereas at the same concentration for cisplatin only 17.80% were at the late apoptotic stage, and for CBD and 5-FU the effects were strong, reaching 100% and 62.55%, respectively. CBD exhibited more noticeable apoptotic results. At 30 µM, cells already reached 78.60% at late apoptotic stage. Overall, the four drugs resulted in a significant increase of the apoptotic cells in a concentration-dependent manner.

## 4. Discussion

In this study, we have applied ECIS to measure frequency- and time-dependent impedance of SCC-25 cell-covered electrodes and to assess subtle changes in cell morphology and micromotion in response to different concentrations of anticancer compounds. The strength of the ECIS method is its capability to follow progressive cellular responses to the challenge of anticancer drugs and to distinguish among a few morphological parameters which contribute to the decrease of impedance. For example, a gradual drop in resistance time course caused by 100 μM cisplatin (green line in Figure 2D) was mainly due to the approximate 25% decrease of Rb (Figure 3B) rather than the change of h (Figure 3C). However, this cytotoxic effect caused significant reduction in micromotion (Figure 4). Exposure of SCC-25 cells to sufficiently high concentration of andrographolide (≥30 μM), CBD (≥10 μM), cisplatin (≥100 μM), or 5-FU (≥100 μM) causes cytoplasmic shrinkage and cell shape changes, leading to different levels of decrease in the overall resistance time courses (Figure 2). Loss of cell–cell contacts (Rb decrease) and/or cell–substrate contacts (h increase) is the general cause for these apoptosis-induced changes in cell morphology. The simultaneous monitoring of Rb and h is a typical example of versatility which the ECIS technique can provide.

In general, apoptosis-induced cytoplasmic shrinkage is concomitant with reduction of cell micromotion. As shown in Figure 2, Figure 3 and Figure 4, exposure of SCC-25 cells to CBD (at 30 or 100 μM) or 5-FU (at 100 μM) causes overall resistance time course drop, Rb decrease, h and Cm increase, and Var32 decrease. However, a large increase in Var32 value is observed after exposure of cells to 30 μM andrographolide, while the overall resistance slightly drops, Rb decreases, h increases, and Cm has almost no change. The reason for the large increase in Var32 upon exposure of SCC-25 cells to 30 μM andrographolide is not known. Although overall resistance and micromotion represent different features of the cells, the large Var32 value obtained from andrographolide at 30 μM is possibly related to the alternations in cell–cell contacts. Further experiments using sub-confluent cell layers will sort out the source of these enhanced impedance fluctuations.

In parallel with the ECIS monitoring of morphological changes associated with apoptosis induced by anticancer compounds, biochemical assays were performed to support these findings. Previous studies have shown the inhibition of invasion and migration in other cancer cells under treatment with andrographolide [33,34]. CBD has also shown promising results as an anticancer drug in recent studies. Cell viability assessed by alamarBlue was evaluated to attain the significant inhibitory concentrations of all four drugs. Interestingly, the profile of the cell viability (%) over different concentrations (Figure 5) is similar to that of Var32 ratio (Figure 4), indicating a correlation between these two measures. Following the anti-proliferative activity of the cells after drug treatment, we confirmed if these effects were dependent on apoptosis through Annexin V stain and flow cytometry. From our results, we observed the same concentration-dependent increase in the apoptotic cells for all four drugs in comparison to the control and DMSO groups. These biochemical findings are in agreement with the ECIS results. Taken together, our results show that in terms of apoptosis-induced changed in cell morphology, micromotion, and cell viability, CBD shows the most cytotoxic effect on SCC-25 cells among the four compounds. Furthermore, this study also presents a useful impedance-based cellular assay for evaluating chemotherapeutic agents and cancer research. ECIS is prominent and sensitive on physiological and pharmacological tests, and is suitable for emerging drugs as the initial test platform.

## 5. Conclusions

In this study, we applied ECIS to investigate the cytotoxic effects of four anticancer drugs on SCC-25 cell morphology and micromotion. These measurements exhibited a concentration-dependent response in the SCC-25 cells treated with all four drugs and the results are correlated with biochemical assay. These data suggest that CBD may be a potentially useful therapeutic treatment for oral cancer following an apoptotic pathway. Furthermore, the ECIS method provides a versatile and sensitive approach to perform pharmacological testing.

## Supplementary Materials

The following are available online at https://www.mdpi.com/1424-8220/20/9/2632/s1, Figure S1: Normalized resistance time course recorded 24 h after the addition of various concentrations of (A) andrographolide, (B) CBD, (C) 5-FU, and (D) cisplatin to confluent SCC-25 cells. The final concentrations of each compound were 10 μM (red), 30 μM (blue), 100 μM (green), 300 μM (yellow), and control (black). Each curve consists of 2048 data points taken at 1 s sampling time. Here, the total 2048 data points in each curve were used to calculate its Var32 value.
