# Supplementary material for: Electrochemical Assessment of Anticancer Compounds on the Human Tongue Squamous Carcinoma Cells
Source: Sensors (Basel). 2020 May 5;20(9):2632. doi: 10.3390/s20092632 (PMC7249152; doi:10.3390/s20092632)
Supplement: Supplementary file 1 [file sensors-20-02632-s001.pdf]

sensors

MDPI

Supplementary Materials

Electrochemical Assessment of Anticancer Compounds on the Human Tongue Squamous Carcinoma Cells

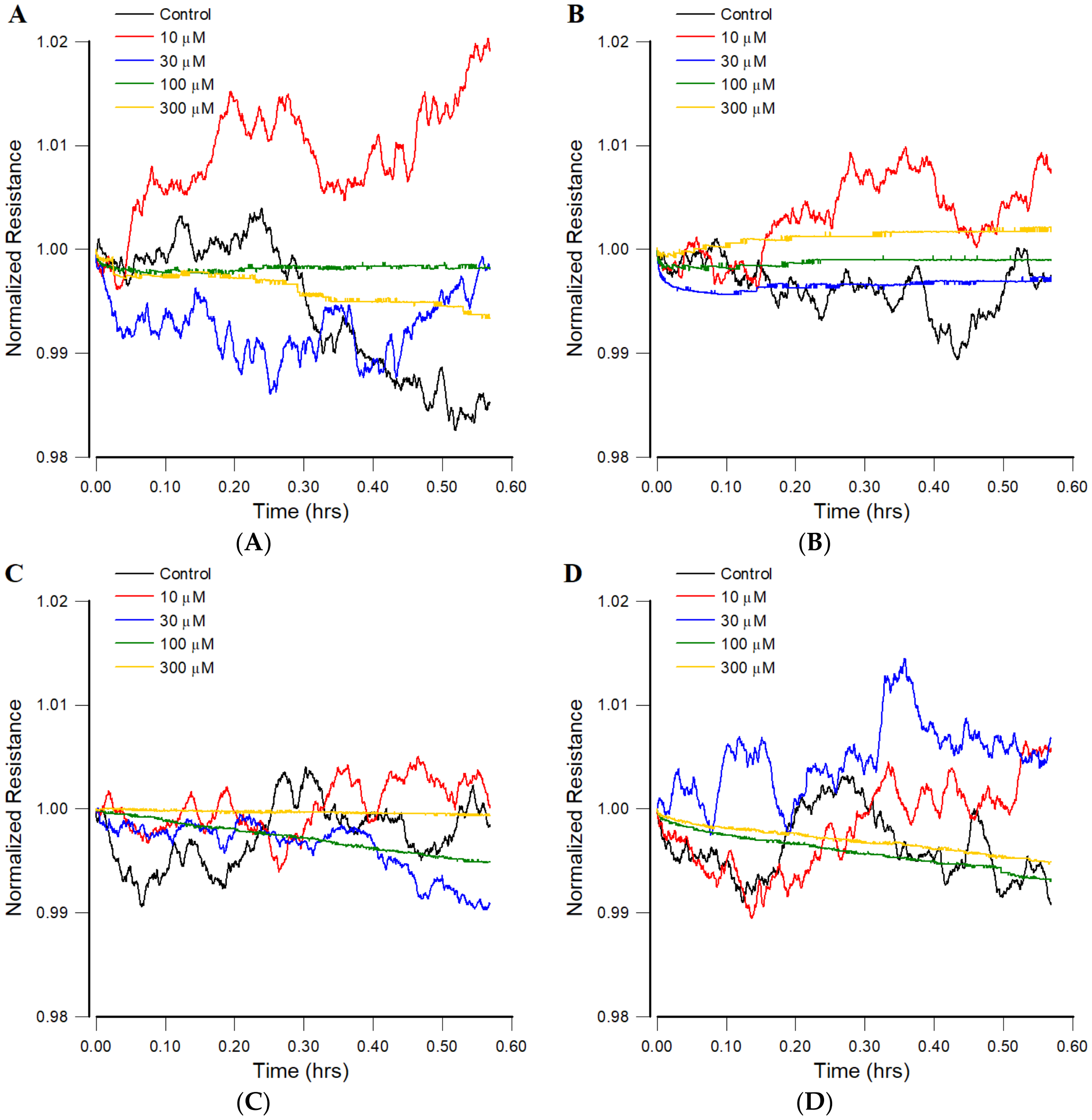

Figure S1. Normalized resistance time course recorded 24 h after the addition of various concentrations of (**A**) andrographolide, (**B**) CBD, (**C**) 5-FU, and (**D**) cisplatin to confluent SCC-25 cells. The final concentrations of each compound were 10 µM (red), 30 µM (blue), 100 µM (green), 300 µM (yellow), and control (black). Each curve consists of 2048 data points taken at 1-s sampling time. Here the total 2048 data points in each curve were used to calculate its Var32 value.
